# Phenotypic plasticity is broadly adaptive across an elevation gradient in the Cutleaf Monkeyflower

**DOI:** 10.1111/nph.71365

**Published:** 2026-06-19

**Authors:** Jill M. Love, Kathleen G. Ferris

**Affiliations:** ^1^ Department of Ecology and Evolutionary Biology Tulane University 6823 St. Charles Avenue New Orleans LA 70118 USA

**Keywords:** adaptive plasticity, genotypic selection analysis, leaf lobing, local adaptation, phenotypic plasticity, reciprocal transplant

## Abstract

- Phenotypic plasticity is a key mechanism by which organisms cope with environmental heterogeneity, but its evolutionary consequences depend on how plastic responses align with the broader adaptive landscape.
- We tested whether plasticity in leaf shape is associated with differential fitness across an elevational gradient in *Mimulus laciniatus*, an annual wildflower endemic to montane California. Using a reciprocal transplant experiment and recombinant inbred lines (RILs) previously phenotyped for plasticity in controlled conditions, we measured variation in survival and fecundity in native low‐ and high‐elevation habitats.
- Based on previous work, we expected to find selection against leaf shape plasticity at low elevations and selection for leaf shape plasticity in the high‐elevation direction (increased leaf lobing under long‐day conditions) at high elevations. However, plastic RILs outperformed nonplastic genotypes at all elevations. We found that RIL genotypes exhibiting high‐elevation plasticity were most likely to set seed at high elevations, but low‐elevation plasticity (increased leaf lobing under short‐day conditions) was associated with greater seed number (fecundity) across elevations.
- This pattern suggests that plasticity is broadly beneficial across *M. laciniatus*'s geographic range and that the direction of plasticity is subject to locally varying selection.

## Graphical abstract

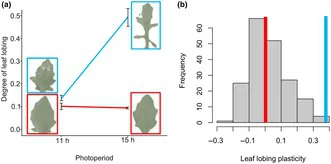

## Introduction

In evolutionary ecology, many discussions have centered around how phenotypic plasticity affects an organism's reproductive success across heterogeneous environments. Phenotypic plasticity, the ability of a single genotype to express multiple phenotypes depending on environmental conditions, offers organisms a degree of flexibility that may promote survival and reproduction when conditions shift (Bradshaw, 1965; West‐Eberhard, 2003). By buffering against environmental variability, plasticity may enhance fitness in unpredictable or novel habitats, making it a potentially adaptive trait in the face of climate change or spatial heterogeneity (Via *et al*., 1995; Ghalambor *et al*., 2007). However, plasticity is not universally beneficial. It can incur physiological or genetic costs, such as resource allocation trade‐offs, slower development, or increased susceptibility to herbivory or pathogens due to mismatched trait expression (Dewitt *et al*., 1998; Auld *et al*., 2010). Additionally, plastic responses beneficial in one environment may prove maladaptive in another, particularly if environmental cues are unreliable or plasticity limits local specialization (Gomulkiewicz & Kirkpatrick, 1992). These context‐dependent effects of plasticity make it difficult to generalize about its fitness consequences.

Despite decades of theoretical attention, empirical data on how plasticity influences fitness in the wild remain sparse, and foundational questions remain unanswered. This is partly due to logistical challenges: Testing plasticity's fitness effects requires genotypic replication across contrasting environments, a task constrained by the difficulty of producing and transplanting clonal individuals into natural settings. Only a handful of studies have surmounted these challenges to provide empirical tests of selection on plasticity. These studies demonstrate that selection on plasticity can be quantified, is frequently environment‐dependent, and that its fitness consequences vary with ecological context and the spatial scale of environmental heterogeneity. Scheiner & Callahan (1999) provide a quantitative framework for estimating selection on reaction norms, while reciprocal transplant experiments with genotypic replication show both adaptive and constrained plastic responses. For example, Donohue *et al*. (2000) found density‐ and site‐dependent selection consistent with adaptive plasticity in shade‐avoidance traits in *Impatiens capensis*, whereas Dechaine *et al*. (2007) detected significant costs of plasticity for several traits in field‐grown *Brassica rapa* recombinant inbred lines (RILs). Studies in heterogeneous serpentine landscapes further show that spatial environmental scale shapes selection on plasticity, with selection favoring plasticity in finer‐scale environments (Baythavong & Stanton, 2010; Baythavong, 2011). Longer term and higher dimensional approaches reinforce these patterns: Anderson *et al*. (2021) documented selection favoring plasticity in leaf traits in *Vaccinium elliottii*, Hamann *et al*. (2025) detected selection on gene expression plasticity in rice across wet and dry field environments and found no evidence that adaptive plasticity was costly, and Walter *et al*. (2026) showed that scale‐dependent genetic differences in plasticity predict fitness along an elevational gradient. Further research will build upon these studies to develop a deeper understanding of how plasticity is shaped by selection in complex and dynamic field environments.

Understanding plasticity's role in local adaptation is especially interesting in environments that present steep ecological gradients. Altitudinal gradients in particular provide powerful natural laboratories to test for selection on phenotypic traits across contrasting environments because climate, abiotic stressors, and biotic interactions shift dramatically from low to high elevation within relatively short geographic distances (Körner, 2007; Halbritter *et al*., 2018). Across elevation, changes in temperature, growing season length, photoperiod, and moisture availability can create divergent selection pressures, driving population‐level trait divergence. Plants are powerful models for studying adaptive plasticity because they are sessile organisms that must cope with environmental heterogeneity throughout their life cycle, often through developmentally flexible traits (Bradshaw, 1965; Sultan, 2000; Nicotra *et al*., 2010). Annual plants are useful for evolutionary studies of plasticity because they experience only one reproductive season, allowing fitness to be measured comprehensively across environments in a single year (Schlichting & Smith, 2002; Donohue, 2003). In addition, their short generation times enable selection experiments across multiple environmental gradients. *Mimulus laciniatus* A. Gray, the Cutleaf Monkeyflower, is a valuable model species for plasticity research, as it is a self‐fertilizing, short‐lived annual plant endemic to the Sierra Nevada (California, USA), growing across an elevational range from *c*. 1000 to 3000 m. In montane regions such as the Sierra Nevada, lighter snowpack and earlier spring warming in low‐elevation populations mean these plants experience shorter days and higher temperatures, while high‐elevation populations are characterized by cooler temperatures, higher snowpack, and shorter growing seasons under longer late‐spring/early‐summer photoperiods. Notably, snowpack coefficient of variance in this region of the Sierra Nevada is markedly higher at low elevation when compared to high elevation (Supporting Information Fig. S1; California Department of Water Resources, 2026). These stark contrasts create different ‘experienced photoperiods’ and levels of environmental heterogeneity, which could create divergent selection pressures that favor different values of plasticity depending on elevation. In this study, we performed a reciprocal transplant experiment between native low‐ and high‐elevation environments of *M. laciniatus*, to test for selection on replicate recombinant genotypes varying in plasticity. This approach allows us to disentangle local adaptation from plastic responses by measuring trait–fitness associations at both native and non‐native altitudes (Clausen *et al*., 1941; Ågren & Schemske, 2012).

In the genus *Mimulus*, trait divergence across environmental gradients is well‐documented, with evidence of selection on phenology, growth, and stress tolerance (Lowry *et al*., 2008; Hall *et al*., 2010; Sheth & Angert, 2016; Ferris & Willis, 2018; Kooyers *et al*., 2019; Tataru *et al*., 2023). Previous work in *M. laciniatus* demonstrated that populations differ in mean trait values and the degree of leaf shape plasticity across elevation (Love & Ferris, 2024). In *M. laciniatus*, high‐elevation populations exhibit greater leaf lobing and leaf shape plasticity in response to increased photoperiod (high‐elevation plasticity), whereas low‐elevation populations exhibit little leaf shape plasticity, or plasticity in the opposite direction in which leaves become more lobed under shorter photoperiods (low‐elevation plasticity). This difference corresponds to elevation‐specific environmental cues, specifically the timing of snowmelt causing high‐elevation populations to germinate and develop later under longer photoperiods closer to the summer solstice than low‐elevation *M. laciniatus*. These repeated clines in plasticity expression across the species' range suggest that plasticity itself may respond to locally varying selection, potentially contributing to local adaptation. While Love & Ferris (2024) provided strong evidence for divergent plastic responses in controlled environments, it was still unclear whether those plasticity differences influence fitness in the wild. This study builds on this previous work, using a newly created RIL population and field‐based approach to ask whether plasticity in *M. laciniatus* contributes to local adaptation across elevations.

By measuring multiple phenotypic traits, we capture a fuller picture of how selection acts across different functional axes of plant growth. Given well‐established links between plant size, height, flowering time, and fitness in annual species, we predict that RILs exhibiting larger size, taller growth, and earlier flowering will have higher fitness across elevation (Mitchell‐Olds, 1996; Stanton *et al*., 2000; Hall & Willis, 2006; Franks *et al*., 2007; Wadgymar *et al*., 2017). We also predict that the direction and magnitude of selection on plastic genotypes will vary based on environmental context, as demonstrated in field studies across plant taxa. For example, in *I. capensis*, selection favored plastic shade‐avoidance traits in dense understory sites, but not in open, low‐density habitats (Donohue *et al*., 2000), demonstrating that the fitness benefits of plasticity may emerge only under particular environmental constraints. By contrast, in *B. rapa*, plasticity incurred detectable fitness costs under high‐density conditions (Dechaine *et al*., 2007). Here, we examine the adaptive significance of leaf shape plasticity in *M. laciniatus* as leaf shape is known to be under positive selection in the species' unique rocky outcrop habitat (Ferris & Willis, 2018). Leaf shape is known to influence key plant physiological processes (Nobel, 1999). More highly lobed leaves reduce boundary layer thickness, improving convective heat loss in hot environments (Givnish & Vermeij, 1976; Givnish, 1979; Gurevitch & Fox, 2006; Nicotra *et al*., 2011). Lobed leaf morphology may also enhance hydraulic efficiency and reduce resistance to water transport, allowing improved transpiration in dry or otherwise stressful environments (Nicotra *et al*., 2011). However, these functional advantages are environment‐dependent, and leaf shapes that enhance cooling and transpiration in warm, dry environments may be less advantageous in cooler or less stressful environments. As a result, no single leaf morphology is expected to be optimal across the full range of environments experienced by *M. laciniatus*. Plasticity in leaf lobing may therefore be under selection because it allows individuals to express locally favored leaf shapes in response to environmental conditions, contributing to locally adaptive trait expression across elevations.

To test for locally varying selection on phenotypic plasticity, we generated a RIL population from low‐ and high‐elevation *M. laciniatus* genotypes exhibiting different degrees of leaf shape plasticity. Because plasticity segregates randomly with respect to other traits in the RIL population, RILs allow us to test whether plasticity itself is broadly or locally adaptive across an elevational gradient of *M. laciniatus* native habitat. If plasticity is broadly beneficial in buffering against environmental variation in this species, we expect positive selection for more plastic RIL genotypes across both high‐ and low‐elevation sites. However, if plasticity imposes costs or only confers advantages under specific conditions (i.e. is locally adaptive with trade‐offs), the direction and magnitude of selection on plastic genotypes will vary depending on the environmental context. Previously observed plasticity clines in Love & Ferris (2024) suggested that plasticity varied in a locally adaptive manner, and therefore, we expected to find selection against plasticity in low‐elevation environments and selection for high‐elevation plasticity (increased leaf lobing in long days) at high elevations. By measuring genotypic selection on 4950 individuals across a reciprocal transplant between five common gardens, we tested whether plasticity in leaf shape, along with mean variation in leaf shape, plant height, and flowering time, is locally adaptive across *M. laciniatus*'s altitudinal range.

## Materials and Methods

### Creation of recombinant inbred lines

To measure phenotypic selection on a genetically replicable population, we generated RILs that segregate in leaf shape plasticity expression. We created a population of 225 *Mimulus laciniatus* A. Gray RILs by crossing a low‐elevation, nonplastic *M. laciniatus* genotype (HEH 20; Hetch Hetchy, CA; elevation 1397 m) with a high‐elevation, highly plastic *M. laciniatus* genotype (WLF 72; White Wolf Campground, CA; elevation 2395 m) to make an F_1_ hybrid (Fig. 1a). The native WLF and HEH populations are *c*. 23.13 km apart. We then self‐fertilized a single F_1_ hybrid to create 500 F_2_ individuals, self‐fertilizing each line to the F_8_ generation (Pollard, 2012). Over the course of RIL creation, we lost 55% of lines to inbreeding depression. A crossing diagram depicting the details of RIL design can be found in Fig. S2. Due to logistical constraints and rapid drying of our field transplant sites, we were unable to consistently obtain high‐quality leaf shape measurements across all genotypes and elevations in field conditions without damaging the plants and affecting fitness. As a result, we used phenotypic data from a common garden growth chamber experiment to represent genotypic values of trait plasticity, and data from a separate common garden glasshouse experiment to quantify genotypic mean traits in each RIL. These common garden experiments are described in detail in the next paragraph. This approach aligns with established genotypic selection frameworks that estimate selection on traits using average trait values per genotype (Rausher, 1992; Stinchcombe & Rausher, 2001). Due to logistical constraints mentioned previously, our approach differs slightly in that trait means were measured in a common environment while fitness was measured in the field. This design enables us to evaluate whether trait plasticity, as an inherent property of a genotype, correlates with fitness in natural environments.

**Fig. 1:**
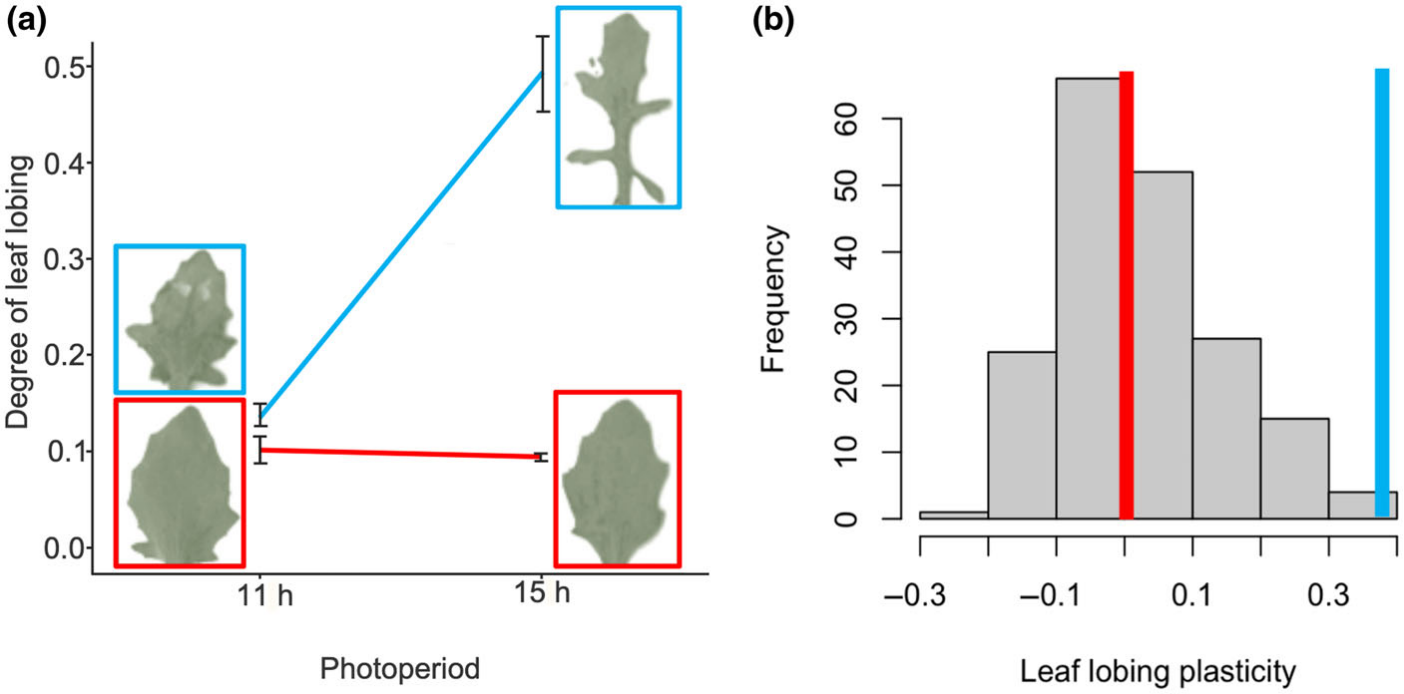
Leaf shape plasticity expression is variable in *Mimulus laciniatus*. (a) Leaf shape reaction norms of low‐ (red) and high‐elevation (blue) parental genotypes used in recombinant inbred line (RIL) creation in low (11 h) and high‐elevation (15 h) photoperiods. Error bars represent SE. (b) Distribution of leaf lobing plasticity expression in the RIL population (gray bins) relative to genotypic plasticity values of the low‐ and high‐elevation parental lines used in RIL creation.

### Measuring genotypic values of mean and plastic RIL traits

To measure the genotypic value of plasticity expressed by each genetic line, we grew three replicates of each RIL and parental genotype under 11‐ and 15‐h photoperiods in a Conviron growth chamber and compared them with mean leaf shape values measured in a separate glasshouse experiment (16‐h photoperiod) as described in Love & Ferris (2024). This design avoids the statistical artifact of regression to the mean (Gunderson & Revell, 2022) because mean trait values and plastic responses are estimated from independent datasets, preventing spurious correlations driven by shared measurement error rather than true genotypic plasticity. On the day of first flower, we recorded flowering time, plant height, and collected the first true leaf for leaf lobing analysis in ImageJ (Ferris *et al*., 2015). Briefly, the lobing index is a metric from zero to one, calculated by dividing the difference between the true area of the leaf and the larger area of the leaf's convex hull by the area of the convex hull. Plasticity per genotype was calculated as the mean 15‐h phenotype subtracted from the mean 11‐h phenotype; the distribution of RIL leaf shape plasticity is displayed in Fig. 1(b). The expression of leaf shape plasticity in the RILs is transgressive relative to the low‐elevation parent. We found significantly more negative values of plasticity than our low‐elevation parent, which had near‐zero plasticity. We are unsure of the mechanisms responsible for this transgressive segregation, but in our previous common garden experiment of *M. laciniatus* from across the species’ range, many low‐elevation genotypes had negative values of plasticity, meaning these genotypes developed more lobed leaves under short days (Love & Ferris, 2024). Therefore, we consider RILs with a negative value of plasticity to have low‐elevation‐like plasticity.

### Field reciprocal transplant experiment

For our field reciprocal transplant experiment in 2024, RIL seeds were planted in 78‐cell trays with Fafard 4B soil, kept moist and dark in stratification at 4°C for 10 d. Trays were then moved to the UC Merced glasshouse and misted daily until germination. Plants stayed in the glasshouse for 7–10 d before transporting to field sites for planting. Glasshouse germination occurred twice during the experiment, for low‐elevation sites on 24 March and high‐elevation sites on 28 May. We chose five common garden locations in native *M. laciniatus* habitat within Yosemite National Park, CA – three at low elevation (Lower Mariposa Grove (LMG), Turtleback Dome (TRT), and Tunnel View (TNL)) and two at high elevation (Yosemite Creek (YCN) and Olmsted Point (OPN)) (Table 1). Planting began after snow first melted and native *M. laciniatus* began to germinate. To plant each reciprocal transplant site, all native angiosperm plants were first cleared within each block, then we transplanted four replicates of each RIL genotype and 100 replicates of each parental genotype at the cotyledon stage into 44 incomplete randomized blocks (25 randomly assigned individuals per block in 5 × 5 array) for a total of 1100 plants per site (Ferris & Willis, 2018; Tataru *et al*., 2023; Dong *et al*., 2025). One low‐elevation site, TNL, had half as many blocks and replicates. We utilized small block sizes due to the heterogeneous nature of *M. laciniatus'* native habitat, which consists of small patches of moss atop exposed rock. No site was larger than 6 × 6 m in area. One week post planting, dead seedlings were replaced to account for transplant shock. Low‐elevation planting finished on 11 April (except TNL, which due to strong snowmelt runoff finished on 4 June) and high‐elevation on 11 June. Sites were monitored every 3 d for survival and flowering. On the date of first flower, plant height was measured. After plants senesced, we collected all fruits and counted total seed number per plant as our fitness metric.

**Table 1:** Geographic information for each reciprocal transplant site.

Site	Elevation (m)		GPS coordinates
Tunnel View (TNL)	Low	1347.0	37.71482, −119.67715
Turtleback Dome (TRT)	Low	1485.2	37.71594, −119.70528
Lower Mariposa Grove (LMG)	Low	1625.1	37.49959, −119.62682
Yosemite Creek (YCN)	High	2325.6	37.84369, −119.57309
Olmsted Point (OPN)	High	2563.6	37.80720, −119.48636

Sites were grouped into low‐ or high‐elevation categories, with exact elevations and GPS locations provided.

### Statistical analyses

#### Environmental variation

All statistical analyses were performed in R v.4.5.1 (R Core Team, 2025). To quantify how survival and environmental variation within each site were associated, we manually collected weekly block‐level environmental data on soil moisture (Dynamax SM150 Soil Moisture Sensor) and soil surface temperature (laser thermometer). We used linear mixed‐effects models with the nlme package in R (Pinheiro *et al*., 2023) to model survival, calculated weekly as the proportion of individuals alive within each block, as a function of time, site, soil moisture, and surface temperature (Ferris & Willis, 2018; Tataru *et al*., 2023; Dong *et al*., 2025). Dependent variables were treated as fixed effects, and block was included as a random effect. To account for temporal nonindependence among repeated observations, we incorporated a first‐order autoregressive correlation structure using the *corAR1* function. Our model was fitted using maximum likelihood (ML) to enable comparison among models with different fixed‐effect structures. We used the *dredge* function in the mu‐min package (Bartoń, 2026) to perform model selection. *Dredge* evaluates all possible subsets of the global model and ranks them by Akaike Information Criterion (AIC). We extracted the best‐supported model based on highest AIC. Finally, to assess the contribution of each predictor in the selected model, we conducted a marginal ANOVA using Type III sums of squares.

#### Local adaption and genotypic selection analysis

We analyzed plant survival, fecundity, and total fitness in parental genotypes to test for local adaptation at low‐ and high‐elevation common gardens. Survival was measured as the proportion of individuals from each parental genotype that survived to flowering at each transplant elevation from the number of replicates planted. We calculated average genotypic fecundity in parental genotypes as total seed number divided by the total number of surviving plants for each genotype. Additionally, we calculated parental total fitness as the number of seeds per planted replicate. Correlation matrices of low‐ and high‐elevation traits were created using the corrplot package in R (Wei & Simko, 2021).

To understand the relationship between measured plant traits and fecundity in our RIL population, we performed genotypic selection analysis using general linear models in the R package glmmtmb (Brooks *et al*., 2017; R Core Team, 2025). To analyze RIL data, we included field‐measured traits as well as genotypic means and genotypic plasticity expression from lab‐collected phenotypes as independent variables in our models, and field‐measured seed number per genotype (genotypic fitness) as our dependent variable. Our independent variables encompassed plant height (field and genotypic mean), flowering time (field and genotypic mean), leaf lobing (genotypic plasticity and mean), and leaf area (genotypic plasticity and mean). Because of limited survival in each individual field site, we combined datasets into two groups, low‐elevation and high‐elevation (Table 1), running full models with all traits in each elevation dataset separately. Trait values were standardized using *z*‐score scaling across elevation datasets to allow direct comparison of selection gradients. We acknowledge that, although the RIL design reduces many correlations among traits, plasticity may still be genetically correlated with unmeasured traits due to tight linkage or pleiotropy that is not fully broken up by recombination. As such, observed associations between plasticity and fitness could partially reflect the effects of these correlated traits.

To deal with an excess of zeros in our fitness data, we used a hurdle model approach (Anderson *et al*., 2015; Ferris & Willis, 2018; Tataru *et al*., 2023). We broke the data into two subsets: (1) all individuals that flowered; and (2) all individuals that flowered and produced at least one seed. For data subset 1, we used a multivariate binomial model to assess whether a trait is associated with producing any seed, with a response of either 0 (no seeds) or 1 (one or more seeds) as the dependent variable. For data subset 2, we used a zero‐truncated Poisson regression as a measure of whether each trait is associated with the number of seeds produced, with fecundity as the dependent variable. Fecundity was calculated as the mean seed production per genotype within each site, averaging across individuals replicated among experimental blocks. Because genotypes were distributed across blocks, this approach incorporates block‐level environmental variation into genotype‐level fitness estimates. The low‐elevation variance in fitness explained by block was 0%; at high elevation, the variance was 3.4%, indicating that block‐level heterogeneity had negligible influence on genotype‐level fitness estimates. Using this design, selection gradients represent partial regression coefficients from multiple regression analysis (Lande & Arnold, 1983). All models can be found in Table 2. For both data subsets, we performed additional model selection using *Mu‐MIn* in R to determine the best‐fit model in each elevation environment (Bartoń, 2026), which can be found in Table S1. To visualize results of the zero‐truncated Poisson models, we used the R package visreg (Breheny & Burchett, 2017).

**Table 2:** Binomial and zero‐truncated Poisson models for low‐ and high‐elevation *Mimulus laciniatus* field sites.

Model type	Elevation	Model description	Log(L)	AIC
Binomial	Low	Any seed set ~ field flowering time + field plant height + genotypic leaf lobing plasticity + genotypic leaf area plasticity + genotypic mean plant height + genotypic mean flowering time + genotypic mean leaf lobing + genotypic mean leaf area	−32.0	82.0
	High	Any seed set ~ field flowering time + field plant height + genotypic leaf lobing plasticity + genotypic leaf area plasticity + genotypic mean plant height + genotypic mean flowering time + genotypic mean leaf lobing + genotypic mean leaf area	−37.4	92.9
Zero‐truncated Poisson	Low	Fecundity ~ field flowering time + field plant height + genotypic leaf lobing plasticity + genotypic leaf area plasticity + genotypic mean plant height + genotypic mean flowering time + genotypic mean leaf lobing + genotypic mean leaf area	−46.8	111.5
	High	Fecundity ~ field flowering time + field plant height + genotypic leaf lobing plasticity + genotypic leaf area plasticity + genotypic mean plant height + genotypic mean flowering time + genotypic mean leaf lobing + genotypic mean leaf area	−430.4	878.8

All predictor variables were included in each model, and log‐likelihood (log(L)) and Akaike Information Criterion (AIC) are provided to assess relative model fit.

We were interested in whether phenotypic plasticity confers a broad or locally adaptive advantage. To test whether different directions of leaf shape plasticity were associated with distinct fitness outcomes across both altitudes, we categorized plasticity expression into three groups: low elevation (plasticity < −0.1); none (plasticity ±0.1 from zero); and high elevation (plasticity > 0.1; Fig. 1b). Phenotypically, negative values of leaf shape plasticity (low‐elevation plasticity) indicated that genotypes expressed more deeply lobed leaves under short days, which was the most common direction of expression in low‐elevation *M. laciniatus* genotypes in Love & Ferris (2024). Positive plasticity values (high‐elevation plasticity) occur when genotypes express more deeply lobed leaves under long‐day photoperiods typically only experienced by high‐elevation populations. To understand the direction of selection on plasticity within each low‐ and high‐elevation habitat, we used the R package emmeans (Lenth, 2025) to conduct *post hoc* asymptotic *z*‐tests on our zero‐truncated Poisson models to compare fitness across the three categories of leaf shape plasticity.

## Results

### Soil moisture and temperature significantly influence survival

Survival declined strongly over the course of the season and was shaped by multiple environmental factors (Figs 2, S3; Table 3). As found in previous field experiments in *M. laciniatus* habitat (Ferris & Willis, 2018; Tataru *et al*., 2023; Dong *et al*., 2025), soil moisture (*F*_1158_ = 20.85, *P* < 0.0001; Fig. 2b) and soil temperature (*F*_1158_ = 7.08, *P* = 0.008; Fig. 2c) each significantly influenced survival. Days since planting × soil moisture was the strongest predictor of survival (*F*_1158_ = 316.07, *P* < 0.0001), indicating a sharp temporal decrease in the proportion of surviving plants modulated by soil moisture (Fig. 2a). The time × soil temperature interaction was also significant (*F*_1158_ = 69.04, *P* < 0.0001), showing that the effect of time on survival depended on moisture and temperature conditions.

**Fig. 2:**
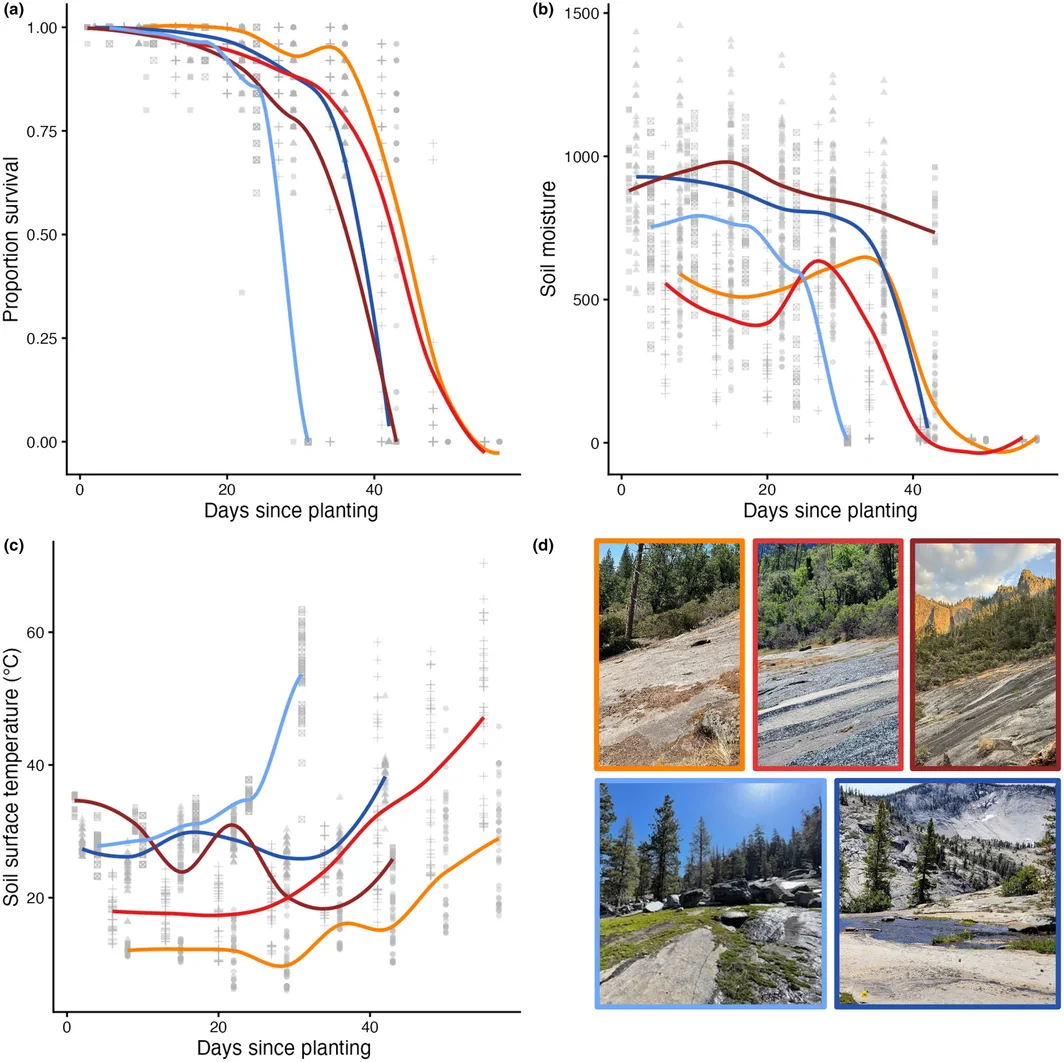
Survivorship curves for experimental *Mimulus laciniatus* plants in low‐elevation (red and orange) and high‐elevation (blue) common gardens. We display (a) proportion survival, (b) soil moisture, and (c) soil surface temperature over time (Table 3). Panel (d) shows a photo of each site.

**Table 3:** Results of ANOVA on a linear model investigating the impact of measured environmental variables on *Mimulus laciniatus* survival along the duration of the reciprocal transplant.

Environmental variable	*F*‐value	*P*‐value
Days since planting × soil moisture	316.07	< 0.0001
Elevation × soil surface temperature	169.62	< 0.0001
Days since planting	91.60	< 0.0001
Days since planting × soil surface temperature	69.04	< 0.0001
Soil moisture	20.85	< 0.0001
Days since planting × elevation	18.30	< 0.0001
Soil surface temperature	7.06	0.008
Elevation	6.42	0.085
Soil moisture × soil surface temperature	2.63	0.105

For all variables but elevation, numDF = 1, denDF = 1158. For elevation, numDF = 1, denDF = 3.

Survivorship curves varied significantly among elevations (*F_1,_*_3_ = 169.62, *P* < 0.0001). We found that elevation × days since planting affected survival (*F_1,_*_3_ = 18.30, *P* < 0.0001). Soil moisture was higher at the beginning of the growing season at high‐elevation sites than it was at two out of three low‐elevation sites (Fig. 2b). The third low‐elevation site, TNL, contained a continuous waterfall that did not dry up even late in the season. Elevation‐level differences also shaped thermal and hydrologic effects. Unexpectedly, we found higher soil temperature in high‐elevation sites than in two out of three low‐elevation sites (Fig. 2c). This is likely because we delayed planting at high‐elevation sites until later in the season due to the timing of snowmelt. However, we see that the seasonal increase in soil temperature was greater at low‐elevation sites than high, agreeing with our expectations. Together, these results highlight that variation in abiotic stressors such as heat and drought exert different selective pressures across elevations.

### The low‐elevation parent has higher fitness across environments

Parental genotypes exhibited distinct reaction norms in survival, fecundity, and total fitness across transplant elevations, but these patterns did not align with classic expectations of local adaptation with trade‐offs. Local adaptation occurs when each genotype has the highest fitness in its home environment relative to a foreign genotype (Kawecki & Ebert, 2004). To assess this, we compared the survival, fecundity, and total fitness of the low‐ and high‐elevation parental genotypes within each elevation. We first assessed differences in viability via parental survival to flowering (Fig. 3a). At low elevation, both parental genotypes exhibited very low survival to flowering, with low‐elevation parents having slightly, but not significantly, greater fitness in their local environment (low parent: 0.8%; high parent: 0%). By contrast, at high elevation, the low‐elevation parent had substantially greater survival (23.0%) than the high‐elevation parent (3.5%), with an estimated odds ratio of 0.10 (95% CI: 0.03–0.25), indicating a roughly 10‐fold difference in survival (Fisher's exact test: *P* < 0.0001). These results show that contrary to predictions under local adaptation with trade‐offs (Kawecki & Ebert, 2004), the low‐elevation genotype outperformed the high‐elevation genotype at both sites and did better at its non‐native site when comparing within genotype across elevations.

**Fig. 3:**
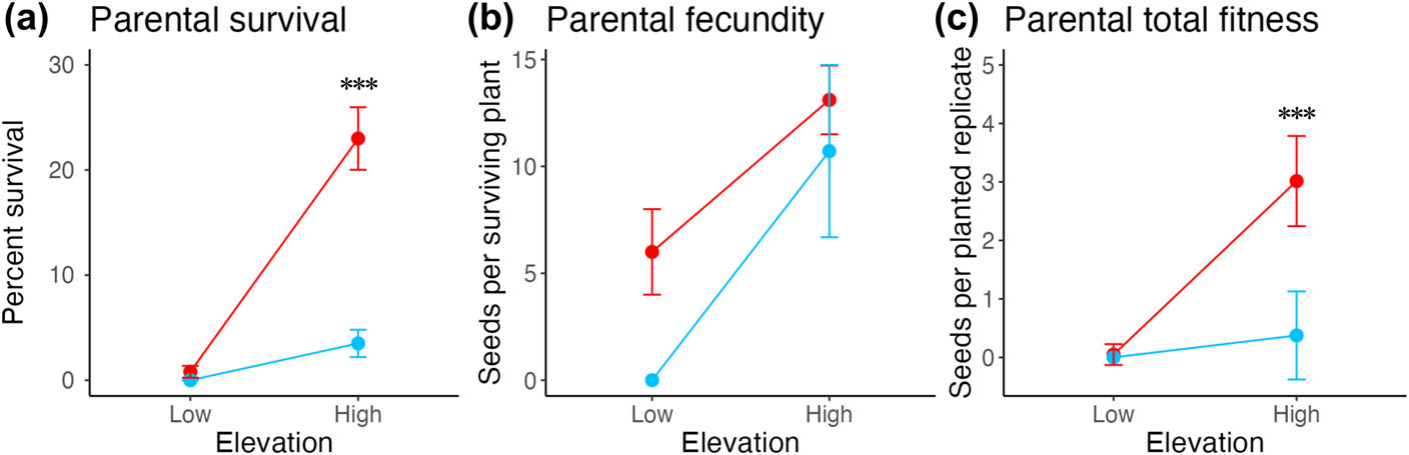
We investigated various fitness metrics in *Mimulus laciniatus* parental genotypes. High‐ (blue) and low‐elevation (red) parental genotype reaction norms in (a) survival, (b) fecundity, and (c) total fitness across experimental garden elevations. Error bars indicate SE and statistical significance is indicated as ***, *P* < 0.01.

Among plants that survived to reproduction, we examined fecundity per surviving individual (Fig. 3b). At low elevation, the low‐elevation parent produced 6.00 seeds per plant on average, but low sample size meant this was not significantly different from the high‐elevation parent's zero seed production (*t*_1_ = 3.0, *P* = 0.205). At high elevation, both genotypes produced similar numbers of seeds, with the low‐elevation parent having slightly higher fecundity (low parent: 13.109 seeds; high parent: 10.714 seeds). This indicates that high‐elevation survival, not fecundity, drives parental differences in fitness (*t*_8_ = 0.552, *P* = 0.596).

Finally, we combined survival and fecundity to estimate total parental fitness as the number of seeds per planted individual (Fig. 3c). At low elevation, both genotypes had near‐zero total fitness, but the low parent had a slightly higher value (low parent: 0.048 seeds, high parent: 0 seeds; Wilcoxon rank‐sum test: *W* = 31 500, *P* = 0.158). At high elevation, the low‐elevation parent produced 3.015 seeds per planted replicate, while the high‐elevation parent produced 0.375 seeds (Wilcoxon rank‐sum test: *W* = 23 934, *P* < 0.0001). Overall, these results suggest that the low‐elevation genotype consistently outperformed the high‐elevation genotype across elevations.

### Both mean and plastic traits are under genotypic selection

To evaluate which traits most strongly predicted the production of any seed set across environments (data subset 1), we used a genotypic selection analysis with multivariate binomial models for low and high elevations (Table 4). These models included combinations of field‐measured traits, genotypic mean traits, and genotypic values of plasticity to evaluate their relative contributions to seed production. We present results from the universal model because it allows direct comparison of selection gradients (*β*) across elevations using the same set of traits. By contrast, model selection alters trait inclusion between habitats, which changes *β* estimates and reduces comparability across environments. Best‐fit models and their selection gradients may be found in Tables S1–S3. A phenotypic correlation matrix of all traits can be found in Fig. S4. At low elevation, only field‐measured plant height was under marginally significant positive selection in the binomial model (*β* = +0.706, *z*_171_ = 1.781, *P* = 0.075). At high elevation, taller field plant height (*β* = +1.903, *z*_169_ = 3.077, *P* = 0.002), earlier field flowering time (*β* = −0.761, *z*_169_ = −2.213, *P* = 0.027), and high‐elevation (positive slope) genotypic leaf lobing plasticity (*β* = +0.762, *z*_169_ = 1.835, *P* = 0.067) all led to a higher likelihood of seed production. These results suggest that binomial selection at high elevation favors taller genotypes with earlier flowering and increased plasticity in the local high‐elevation direction, while at low elevation, only plant height was under weak, marginally significant selection.

**Table 4:** Trait influence on presence of *Mimulus laciniatus* seed production, based on binomial models run on low‐ and high‐elevation datasets.

Trait	Low elevation			High elevation		
	*z*‐value	*P*‐value	Selection gradient (*β*)	*z*‐value	*P‐*value	Selection gradient (*β*)
Field plant height	**1.781**	**0.075**	**+0.706** *	**3.077**	**0.002**	**+1.903** ***
Field flowering time	−1.489	0.137	−0.550	**−2.213**	**0.027**	**−0.761** **
Genotypic leaf lobing plasticity	0.149	0.882	+0.060	**1.835**	**0.067**	**+0.762** *
Genotypic mean plant height	1.471	0.141	+0.476	0.142	0.887	+0.055
Genotypic leaf area plasticity	0.089	0.929	+0.029	−0.216	0.829	−0.080
Genotypic mean leaf lobing	−0.674	0.500	−0.266	−0.854	0.393	−0.290
Genotypic mean flowering time	1.071	0.284	0.358	−1.509	0.131	−0.542
Genotypic mean leaf area	0.109	0.913	0.039	1.114	0.265	+0.437

Selection gradient (*β*) represents the strength and direction of selection on a trait. For low‐elevation traits, numDF = 1, denDF = 171. At high‐elevation, numDF = 1, denDF = 169. Bolded values are statistically significant.

* *P* < 0.10.

** *P* < 0.05.

*** *P* < 0.01.

To identify specific traits under fecundity selection (data subset 2), we fit zero‐truncated Poisson models using genotype‐level data at each site. At low elevation, significant variables in the model included field‐measured plant height and flowering time, genotypic mean plant height and leaf lobing, and genotypic leaf shape plasticity (Table 5; Fig. 4). We found selection for taller plants, measured both as genotypic mean and in the field (field height: *β* = +0.690, *z*_18_ = 2.556, *P* = 0.011, genotypic mean height: *β* = +0.302, *z*_18_ = 1.968, *P* = 0.049), local, low‐elevation leaf shape plasticity (*β* = −0.451, *z*_18_ = −2.432, *P* = 0.015), and selection for greater mean leaf lobing (*β* = +0.326, *z*_18_ = −2.101, *P* = 0.036). At high elevation (Table 5; Fig. 4), field‐measured flowering time was under negative fecundity selection (*β* = −0.282, *z*_96_ = −5.75, *P* < 0.001), while taller field plants (*β* = +0.289, *z*_96_ = 7.07, *P* < 0.001), greater genotypic mean leaf lobing (*β* = +0.088, *z*_96_ = 2.58, *P* = 0.010), and greater plasticity in leaf area were all under positive selection (*β* = +0.115, *z*_96_ = 3.11, *P* = 0.002). Low‐elevation‐like leaf lobing plasticity was also under marginally significant directional selection at high elevation (*β* = −0.070, *z*_96_ = −1.68, *P* = 0.093), which was contrary to the previously observed dominant phenotype in local genotypes at high‐elevation sites (Love & Ferris, 2024).

**Table 5:** Trait influence on number of seeds produced by *Mimulus laciniatus*, based on zero‐truncated Poisson models run on low‐ and high‐elevation datasets.

Trait	Low elevation			High elevation		
	*z*‐value	*P*‐value	Selection gradient (*β*)	*z*‐value	*P*‐value	Selection gradient (*β*)
Field plant height	**2.556**	**0.011**	**+0.690** **	**7.07**	**> 0.001**	**+0.289** ***
Genotypic leaf lobing plasticity	**−2.432**	**0.015**	**−0.451** **	**−1.68**	**0.093**	**−0.070** *
Field flowering time	**−2.453**	**0.014**	**−0.354** **	**−5.75**	**> 0.001**	**−0.282** ***
Genotypic mean leaf lobing	**2.101**	**0.036**	**+0.326** **	**2.58**	**0.010**	**+0.088** ***
Genotypic mean plant height	**1.968**	**0.049**	**+0.302** **	0.34	0.737	+0.012
Genotypic mean flowering time	1.177	0.239	+0.246	−0.55	0.581	−0.201
Genotypic mean leaf area	−1.368	0.171	−0.186	0.02	0.988	+0.001
Genotypic leaf area plasticity	0.854	0.393	+0.145	**3.11**	**0.002**	**+0.115** ***

Selection gradient (*β*) represents the strength and direction of selection on a trait. For low‐elevation traits, numDF = 1, denDF = 18. At high elevation, numDF = 1, denDF = 96. See Fig. 4 for visualization of these results. Bolded values are statistically significant.

* *P* < 0.10.

** *P* < 0.05.

*** *P* < 0.01.

**Fig. 4:**
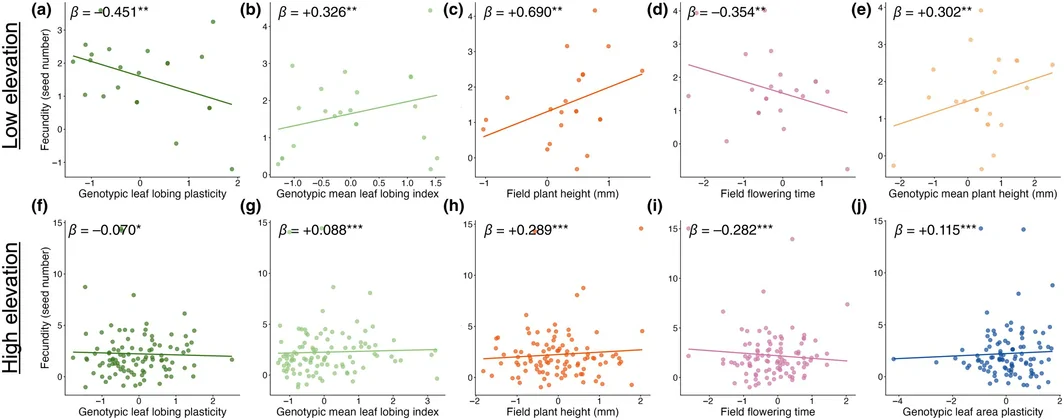
Significant directional fecundity selection gradients for traits at low and high elevations in *Mimulus laciniatus*, using partial residuals from multiple regression of zero‐truncated Poisson models (Breheny & Burchett, 2017). The fitted curves show linear regressions at low and high elevation in statistically significant traits: (a, f) genotypic leaf lobing plasticity, (b, g) genotypic mean leaf lobing, (c, h) field‐measured plant height, (d, i) field‐measured flowering time, (e) genotypic mean plant height, and (j) genotypic leaf area plasticity (Table 5). Nonsignificant selection gradient visualizations can be found in Supporting Information Fig. S5.

### Fecundity selection favors low‐elevation genotype's leaf shape plasticity across elevations

To further probe the relationship between leaf shape plasticity and fecundity within each elevation, we conducted *post hoc* comparisons of estimated marginal means. Pairwise comparisons were conducted using the emmeans package in R on our zero‐truncated Poisson models. We used asymptotic *z*‐tests with degrees of freedom treated as infinite. In low‐elevation gardens, genotypes with low‐elevation plasticity produced significantly more seeds than those with either no plasticity (*β* = +1.059, *z* = 2.512, *P* = 0.032) or high‐elevation plasticity (*β* = +0.631, *z* = 2.549, *P* = 0.029; Fig. 5a). Genotypes with high‐elevation plasticity and no plasticity did not differ in fitness (*z* = 1.208, *P* = 0.449). At high elevation, genotypes with low‐elevation plasticity similarly showed the highest fecundity compared to those with no plasticity (*β* = +0.756, *z* = 4.672, *P* < 0.0001) or high‐elevation plasticity (*β* = +0.238, *z* = 3.350, *P* = 0.002; Fig. 5b). However, at high elevation, both low‐ and high‐elevation leaf shape plasticity significantly increase RIL fitness compared to genotypes with no plasticity, which differed from our results at low elevation (*β* = +0.518, *z* = 3.173, *P* = 0.004).

**Fig. 5:**
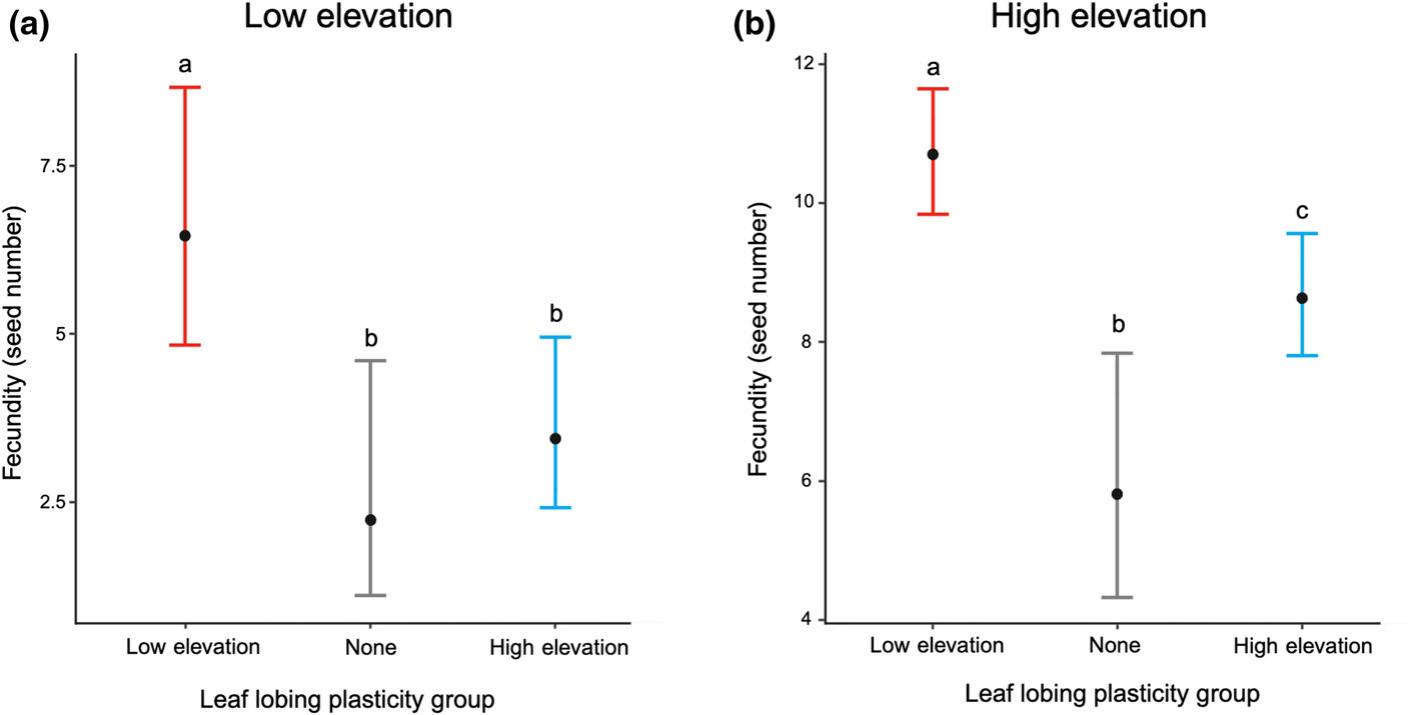
Further examination of leaf shape plasticity's effect on plant fecundity at (a) low‐elevation and (b) high‐elevation in *Mimulus laciniatus*. Asymptotic *z*‐tests revealed significant impacts on fitness based on the direction of plasticity. Black dots indicate estimated marginal means, while error bars show a 95% confidence interval. Low‐elevation leaf shape plasticity increased fecundity, and no plasticity had the lowest fecundity in both common gardens (Table 6).

## Discussion

In this study, we used a reciprocal transplant experiment between low‐ and high‐elevation native *M. laciniatus* habitat in the Sierra Nevada to test for selection on phenotypic plasticity and other traits that differ across elevation. We transplanted RILs that varied in their degree of leaf lobing plasticity, a trait previously shown to exhibit repeated altitudinal clines in this species (Love & Ferris, 2024), between high‐ and low‐elevation gardens and performed genotypic selection analysis. By linking field‐measured fitness to genotypic trait values in 4950 RILs and parental inbred lines across five common gardens, we found that low‐elevation *M. laciniatus* genotypes have the highest fitness at both high and low elevations indicating a potential adaptive lag in which populations from the warm edge of a species' range (low elevation) have higher fitness than the native genotypes in the historically cooler high‐elevation environment (Kooyers *et al*., 2019). In terms of phenotypic selection, we found that plastic RIL genotypes have higher fitness than nonplastic genotypes at all elevations, selection for increased mean leaf lobing consistent with adaptation to rocky outcrop environments, and that the low‐elevation direction of phenotypic plasticity is universally adaptive across *M. laciniatus*'s altitudinal range.

### Moisture and temperature drive survival across the growing season

Our findings reveal a heterogeneous survival landscape shaped by interacting abiotic stressors and provide a mechanistic foundation for interpreting spatial variation in selection on plasticity traits in this system (Fig. 2). Survival of experimental plants declined sharply over the growing season, a pattern driven primarily by time since planting and its interaction with soil moisture, highlighting temporally intensifying drought stress typical of montane Mediterranean systems (Geber & Griffen, 2003; Heschel *et al*., 2004; Franks *et al*., 2007; Angert *et al*., 2011; Ferris & Willis, 2018; Tataru *et al*., 2023). Soil temperature also showed strong main and interactive effects with moisture, supporting a multivariate abiotic stress environment in which warming and desiccation jointly limit survival rather than acting independently (e.g. Sherrard & Maherali, 2006; Wadgymar *et al*., 2018). As the season progresses, the surface temperature increases and may accelerate soil moisture evaporation and plant desiccation, contributing to mortality under compounded abiotic stress. This is particularly relevant in shallow‐rooted environments typical of *M. laciniatus*. Elevation further structured these dynamics: Low‐elevation sites had lower starting temperatures, but a greater increase in temperature throughout the growing season creating lower survival to flowering overall despite one site maintaining high moisture. High‐elevation plants, on the other hand, experienced higher soil moisture but with the addition of mid‐summer heatwaves, which coincided with rapid soil drying. This illustrates that divergent temperature‐moisture trajectories across elevation generate spatially heterogeneous selection regimes. This variation in environmental trajectories across elevation reinforces the importance of considering local environmental context in models of adaptation and plasticity evolution (Ghalambor *et al*., 2007; Richardson *et al*., 2014).

### Parental genotypes show evidence of adaptive lag

Classic local adaptation theory predicts that local genotypes should exhibit higher fitness in their home environments relative to foreign genotypes (Kawecki & Ebert, 2004). Although our reciprocal gardens were located at representative low‐ and high‐elevation habitats, they were not established at the exact native collection sites of the parental genotypes. Consequently, our test evaluates adaptation across elevational environments rather than strict home‐site local adaptation. We found that the low‐elevation *M. laciniatus* genotype has higher total fitness in both its local low‐elevation habitat and at high elevation (Fig. 3). This differs from our predictions, where we expected local vs foreign genotypic advantage in both elevation environments. Our results suggest that the low‐elevation *M. laciniatus* genotype is more robust across habitats rather than locally specialized and that the high‐elevation genotype is maladapted to its current environmental conditions.

Similar patterns of asymmetric performance across environments have been observed in other plant systems and may reflect life‐history trade‐offs or gene flow asymmetries (Hereford, 2009; Wadgymar *et al*., 2017; Kooyers *et al*., 2019). Adaptive lag is one cause of this mismatch across environments, in which the population mean phenotype or genotype remains better suited to past environmental conditions than to the current selective optimum because evolutionary responses have not kept pace with rapid environmental change (Franks *et al*., 2014). For example, Kooyers *et al*. (2019) found that populations of *Mimulus guttatus* from California sites had higher fitness at both high and low‐elevation gardens than local populations in the Cascades, OR. This indicated an adaptational lag in which southern *M. guttatus* populations, historically experiencing higher temperatures and greater drought, had higher fitness in northern gardens than local genotypes under current climate scenarios. Here, the fitness of our high‐elevation *M. laciniatus* may reflect adaptation to past climatic regimes, whereas the low‐elevation genotype, having adapted to historically lower snowpacks and higher temperatures, may have broader physiological tolerance equating to better performance under contemporary warming climatic conditions. This result indicates that future upslope migration may be a likely outcome of adaptation to continued climate change in this species.

### Large plant size, early flowering, and greater leaf lobing are adaptive across *M. laciniatus*'s altitudinal range

Our field study found that both life history and morphological variation are under selection in low‐ and high‐elevation *M. laciniatus* habitats (Fig. 4). Field plant height was under positive selection across elevations, which aligns with long‐standing evidence that increased plant size is associated with higher fitness in annual species due to greater resource acquisition and reproductive potential (Mitchell‐Olds, 1996; Stanton *et al*., 2000). Flowering time was under strong negative selection across both environments. This pattern is seen repeatedly in field‐based annual plant studies including previous studies in *M. laciniatus* (Ferris & Willis, 2018; Tataru *et al*., 2023; Dong *et al*., 2025), in which earlier flowering allows plants to reproduce before the onset of seasonal drought (Hall & Willis, 2006; Franks *et al*., 2007). These consistent patterns across altitudinal habitats suggest that despite environmental heterogeneity across *M. laciniatus*' geographic range, plant size, and timing of reproduction are under uniform selection. This may reflect overarching constraints of the Mediterranean climate regime and *M. laciniatus*' unique rocky outcrop habitat, in which rapid seasonal drying creates a narrow reproductive window (Ferris & Willis, 2018; Fig. 2).

Our previous common garden study (Love & Ferris, 2024) found repeated clines in leaf lobing with high‐elevation populations of *M. laciniatus* having the most highly lobed leaves. This led us to hypothesize that we would find spatially varying selection on mean leaf shape across elevation in our field study. Instead, mean leaf lobing was also under positive selection across low and high elevations. Genotypic selection for greater leaf lobing was in fact stronger in the low‐elevation garden than at the high‐elevation garden (Fig. 4b,g), opposite of our predictions based on clinal variation in natural populations. Phenotypic selection for increased leaf lobing in *M. laciniatus*'s rocky outcrop environment has been found previously (Ferris & Willis, 2018) and is in line with lobed leaf shape's hypothesized function in efficiently regulating leaf temperature and water loss in extreme environments (Nicotra *et al*., 2011). Interestingly, leaf area plasticity was only significant at high elevation. Leaf size is also predicted to modify heat and gas exchange rates between the leaf and environment, with smaller leaves having a reduced boundary layer compared with larger leaves, allowing them to reach air temperature more efficiently through convection (Schuepp, 1993). Given our results, we conclude that both leaf shape and size are likely tied to functional strategies for coping with the abiotic stressors characteristic of rocky alpine environments. These results align with findings from *M. guttatus* as well as wild tomato (*Solanum pennellii*) in which plasticity in leaf morphology has been associated with higher fitness in fluctuating environments (Sultan, 2000; Chitwood *et al*., 2013; Friedman *et al*., 2015).

### Genotypic selection on plasticity direction varies across elevation

The central question of this study was whether, as suggested by our previous work in which we found repeated altitudinal clines in plasticity, plasticity in leaf lobing is locally adaptive with low‐elevation populations being less plastic than high‐elevation genotypes (Love & Ferris, 2024). Our present findings indicate that the fitness consequences of plasticity in leaf lobing are broadly beneficial, while still being environmentally dependent. In our binomial model, testing for the effect of a trait on the probability of setting any seed, we found genotypic selection for high‐elevation leaf shape plasticity in our high‐elevation gardens and no selection on plasticity in low‐elevation gardens (Table 4). While we only tested this hypothesis in a single RIL population, this pattern is consistent with our prediction that plasticity in the direction displayed by the local genotype confers a reproductive advantage. Similar environment‐specific selection on plasticity has been documented in other systems. For example, *I. capensis* populations exhibited density‐ and site‐dependent selection on plastic shade‐avoidance traits, in which plastic individuals performed better only under local light environments (Donohue *et al*., 2000). Likewise, in *B. rapa*, plastic genotypes expressed fitness costs in high‐density environments, demonstrating that selection on plasticity can depend on competitive context (Dechaine *et al*., 2007). These studies along with the results of our binomial selection model illustrate that plasticity can be beneficial only in certain environments, rather than universally adaptive.

In terms of binomial selection on seed production, we found positive selection for high‐elevation plasticity at high elevation (Table 4), implying that increased plasticity in the local direction improves the chance of setting any seed at high elevation. However, our fecundity selection results indicate that RILs with low‐elevation plasticity (negative values) had higher fitness at both low and high elevations (Table 5; Fig. 4a,f). A *post hoc* analysis found that in high‐elevation gardens both low‐ and high‐elevation plasticity RIL genotypes had greater fitness than nonplastic genotypes (Table 6; Fig. 5). Together, these results suggest that selection is operating differently on plasticity at high vs low elevation. Specifically, it seems that the high‐elevation direction of plasticity increases the likelihood of setting seed at high altitudes, while low‐elevation type plasticity increases the number of seeds in low‐altitude environments. We note that our parental lines represent single genotypes from each elevation rather than population‐level samples, and as a result, the variation present in the RIL population may not fully capture the adaptive potential of all native low‐ and high‐elevation populations. To measure genotypic values of mean leaf shape and leaf shape plasticity, we used genotype means derived from controlled growth chamber and glasshouse environments, following from genotypic selection theory to estimate selection on trait values that reflect heritable, environment‐dependent expression (Rausher, 1992; Stinchcombe & Rausher, 2001). One possible limitation of this method is that trait expression was not quantified in the same environments in which fitness was measured. Additionally, our estimates of plasticity were based on photoperiod manipulations, meaning our reaction norms capture only one axis of plasticity relevant to fitness. The normal distribution of plasticity phenotype (Fig. 1a) means that genotypes with the strongest plasticity were also the rarest, which could be due to epistasis, recessive alleles, or a highly quantitative genetic architecture. Our growth chamber treatments quantifying plasticity were designed to simulate photoperiod conditions at the extremes of the species' elevational range, and prior work has shown that these treatments successfully elicit biologically meaningful variation in leaf shape (Love & Ferris, 2024). Our use of replicated RILs across field sites strengthens the inference of selection on genotype‐level traits and plasticity, even if not all traits could be measured directly in the field.

**Table 6:** Influence of leaf lobing plasticity direction on *Mimulus laciniatus* fecundity, based on a *post hoc* test from our zero‐truncated Poisson models at low and high elevation.

Comparison	Low elevation			High elevation		
	*z*‐value	*P*‐value	Selection gradient (*β*)	*z*‐value	*P*‐value	Selection gradient (*β*)
High‐elevation plasticity vs no plasticity	1.208	0.499	+0.433	**3.173**	**0.004**	**+0.518** ***
Low‐elevation plasticity vs no plasticity	**2.512**	**0.032**	**+1.059** **	**4.672**	**< 0.0001**	**+0.756** ***
Low‐elevation plasticity vs High‐elevation plasticity	**2.549**	**0.029**	**+0.631** **	**3.350**	**0.002**	**+0.238** ***

We used asymptotic *z*‐tests with degrees of freedom treated as infinite. Bolded values are statistically significant.

** *P* < 0.05.

*** *P* < 0.01.

Plasticity is most likely to be maintained when environments fluctuate spatially and temporally, such that no single trait value is optimal everywhere (Crispo, 2008; Snell‐Rood & Ehlman, 2021). In high‐elevation environments with unpredictable timing of snowmelt and snowfall, balancing selection may maintain both directions of leaf lobing plasticity based on temporally fluctuating selection gradients. It is possible that temporally variable selection, known to be a persistent force in *M. laciniatus*'s rocky outcrops due to dramatic interannual climatic fluctuations (Tataru *et al*., 2023; Dong *et al*., 2025), maintains genetic variation in plasticity direction. In agreement with this interpretation, genetic variation in plasticity direction does exist within high‐elevation *M. laciniatus* populations such that some high‐elevation genotypes exhibited the low‐elevation direction of plasticity in our previous experiment (Love & Ferris, 2024). Our experiment, performed during an average snowpack year but where snowmelt happened rapidly, may have caught conditions that give low‐elevation plasticity genotypes a universal fecundity advantage. It is also possible that, like our results in the relative fitness of high and low *M. laciniatus* parental genotypes, the lack of local adaptation in plasticity direction at high elevation indicates a mismatch between historical and current selection pressures creating adaptive lag in phenotypic plasticity as well as parental fitness (Kooyers *et al*., 2019). Perhaps as the climate continues to warm and the timing of snowmelt moves earlier, creating exposure to shorter day lengths during the growing season at high elevations, we will see an increase in frequency of low‐elevation plasticity genotypes in high‐elevation *M. laciniatus* populations. Low‐elevation environments may favor local low‐elevation plasticity, in which more lobed leaves are produced under local shorter photoperiods, as a developmentally labile means to produce lobed leaves uniquely found in rocky outcrop *Mimulus* species (Ferris *et al*., 2015).

### Minimal costs of phenotypic plasticity in a native field environment

Many theoretical studies propose that plasticity itself is costly (Via *et al*., 1995; DeWitt, 1998). These costs may occur via the energetic expense of developing or maintaining a plastic response, or through maladaptive mismatches under certain conditions (Auld *et al*., 2010). In our field experiment, we found that leaf shape plasticity was broadly beneficial, enhancing fitness across both elevation environments. However, the adaptive value of plasticity depended on the direction of the trait expression: genotypes expressing low‐elevation plasticity (i.e. negative reaction norms) had the highest fecundity at both low and high elevation, while genotypes with high‐elevation plasticity (i.e. positive reaction norms) only had a higher likelihood of setting any seed at high elevation. This pattern suggests asymmetric selection on plasticity and raises the possibility of context‐dependent costs, as we detected differential selection on three different directions of reaction norm slope (negative, neutral, and positive) in one plastic trait.

Yet, we observed that plastic genotypes performed better than nonplastic genotypes in both environments, and thus did not detect evidence of a cost of plasticity. This aligns with a large body of literature showing that costs of plasticity are difficult to detect in field conditions, only emerging under specific or stressful contexts (Van Kleunen & Fischer, 2005; Callahan *et al*., 2008; Matesanz *et al*., 2010). For example, in *Boechera stricta*, Wagner & Mitchell‐Olds (2018) found that plasticity in defense chemistry was associated with greater fitness across contrasting habitats but did not show consistent selection within any single environment, reinforcing the idea that plastic responses can be beneficial across multiple contexts without strict local adaptation. Empirical tests across taxa often fail to find consistent fitness costs associated with plastic responses (Ghalambor *et al*., 2007; De Lisle & Rowe, 2023). Our results contribute to this literature by demonstrating that, in a median‐snowfall year with a perhaps more relaxed selection regime than drought years, leaf shape plasticity conferred fitness benefits rather than repercussions. Adding to this finding, we saw that the direction of plasticity expression mattered across different environments. Therefore, in *M. laciniatus*, costs likely manifest not from the mechanistic presence of plasticity itself, but from environmental mismatches with the direction of plastic responses.

### Conclusion

Taken together, our findings offer insight into how selection acts on phenotypic plasticity in natural populations. We contribute empirical evidence from a large‐scale reciprocal transplant experiment demonstrating that warm edge genotypes have a universal advantage across *M. laciniatus*'s altitudinal range indicating a potential adaptive lag, that plastic genotypes are universally more fit than nonplastic genotypes, and that local directions of plasticity are sometimes, but not always, more beneficial. The adaptive significance of phenotypic plasticity has long been debated in evolutionary ecology, in recent years becoming especially prominent considering the role plasticity could play in the race against ongoing global climate change. We find that plasticity in one trait is adaptive, depending on the environmental context. As high‐elevation plant populations continue to experience historically earlier springs, growing under warmer temperatures and shorter photoperiods, we may see an upslope migration of leaf shape plasticity genotypes in *M. laciniatus*. This work advances understanding of the evolutionary dynamics of plasticity in heterogeneous landscapes and highlights the value of integrative approaches that link phenotypic plasticity and fitness in the wild.

## Competing interests

None declared.

## Author contributions

JML and KGF conceptualized the research, funded the research and wrote the manuscript. JML performed all experiments, fieldwork, data collection and data analysis. KGF provided supervision and other resources.

## Disclaimer

The New Phytologist Foundation remains neutral with regard to jurisdictional claims in maps and in any institutional affiliations.

## Supporting information


**Fig. S1** Snowpack variation in the Sierra Nevada at low and high elevation.
**Fig. S2** Crossing design of *Mimulus laciniatus* leaf shape plasticity recombinant inbred line population.
**Fig. S3** Weekly *Mimulus laciniatus* survival data plotted against soil surface temperature and soil moisture.
**Fig. S4** Correlation matrices of all *Mimulus laciniatus* traits included in genotypic selection models for low and high elevation.
**Fig. S5** Nonsignificant directional fecundity selection gradients for remaining traits in *Mimulus laciniatus* at low and high elevations.
**Table S1** Comparison of best‐fit binomial and zero‐truncated Poisson models for low‐ and high‐elevation *Mimulus laciniatus* field sites after model selection.
**Table S2** Trait influence on *Mimulus laciniatus* survival to seed production (best‐fit models).
**Table S3** Trait influence on *Mimulus laciniatus* number of seeds produced (best‐fit models).
Please note: Wiley is not responsible for the content or functionality of any Supporting Information supplied by the authors. Any queries (other than missing material) should be directed to the *New Phytologist* Central Office.

## Data Availability

Data and code are available on GitHub at the following repository: https://github.com/jill‐love/Love‐Ferris‐2026.
