# Supplementary material for: Phenotypic plasticity is broadly adaptive across an elevation gradient in the Cutleaf Monkeyflower
Source: New Phytol. 2026 Jun 19;252(4):1645–59. doi: 10.1111/nph.71365 (PMC13669800; doi:10.1111/nph.71365)
Supplement: Supplementary file 1 [file NPH-252-1645-s001.docx]

New Phytologist Supporting Information
Article title: Phenotypic plasticity is broadly adaptive across an elevation gradient in the Cutleaf Monkeyflower
Authors: Jill M. Love, Kathleen G. Ferris
Article acceptance date: 1 June 2026

**Supplemental Figures**

Figure S1. Snowpack in the Sierra Nevada fluctuates annually. Panel **(a)** shows snowpack at April 1 in four monitored central-Sierra sites from 2000-2026, red sites being low-elevation and blue sites being high-elevation. Panel **(b)** shows the coefficient of variation (CV) in April 1 snowpack across years; low-elevation sites have a higher CV than high-elevation sites since 2000. Data from California Department of Water Resources (2026).

Figure S2. Crossing design of *M. laciniatus* leaf shape plasticity RIL population.

(a)

(b)

Figure S3. Weekly *M. laciniatus* survival data plotted against weekly (a) soil surface temperature and (b) soil moisture.

Figure S4. Correlation matrices of all *M. laciniatus* traits included in genotypic selection models for low- and high-elevation. Blank squares indicate statistically insignificant (p > 0.05) correlations. At low-elevation, field N = 121 plants; mean N = 2054 plants; plastic N = 1844 plants. At high-elevation, field N = 421 plants; mean N = 1640 plants; plastic N = 1472 plants.

Figure S5. Non-significant directional fecundity selection gradients for remaining traits in *M. laciniatus* at low and high-elevations, using partial residuals from multiple regression of zero-truncated Poisson models [(Breheny & Burchett, 2017)](https://paperpile.com/c/y4S6ru/Qy6N). The fitted curves show linear regressions at low and high-elevation in statistically significant traits: **a&d)** genotypic mean flowering time, **b&e**) genotypic mean leaf area, **c**) genotypic leaf area plasticity, and **f**) genotypic mean plant height (Table 5).

**Supplemental Tables**

Table S1. Comparison of best-fit binomial and zero-truncated Poisson models for low- and high-elevation *M. laciniatus* field sites after model selection using MuMIn. Log(L) and AIC are provided to assess relative model fit.

| Model type | Elevation | Model description | Log(L) | AIC |
| --- | --- | --- | --- | --- |
| Binomial | Low | any seed set ~ field flowering time + field plant height | -33.5 | 73.0 |
|  | High | any seed set ~ genotypic mean flowering time + field flowering time + field plant height | -38.2 | 86.5 |
| Zero-truncated Poisson | Low | fecundity ~ field plant height + genotypic mean plant height + genotypic leaf lobing plasticity | -50.5 | 109.0 |
|  | High | fecundity ~ field plant height + genotypic mean plant height + genotypic leaf lobing plasticity + field flowering time + genotypic leaf area plasticity + genotypic mean leaf lobing | -445.5 | 902.9 |

Table S2. Trait influence on *M. laciniatus* survival to seed production (best-fit models). For low-elevation traits, numDF = 1, denDF = 171. At high-elevation, numDF = 1, denDF = 169. Variables with n.s. were not included in the best-fit model and therefore do not have a selection gradient to report. * p < 0.10, ** p < 0.05, *** p < 0.01

|  | Low-elevation | | | High-elevation | | |
| --- | --- | --- | --- | --- | --- | --- |
| Trait | z-value | p-value | Selection gradient (β) | z-value | p-value | Selection gradient (β) |
| Field plant height | 1.628 | 0.104 | +0.578 | **3.050** | **0.002** | **+1.689***** |
| Field flowering time | -1.457 | 0.145 | -0.489 | **-2.189** | **0.029** | **-0.736**** |
| Genotypic leaf lobing plasticity | n.s. | n.s | n.s. | **1.76** | **0.078** | **+0.675*** |
| Genotypic mean flowering time | n.s. | n.s | n.s. | -1.553 | 0.121 | -0.500 |

Table S3. Trait influence on *M. laciniatus* number of seeds produced (best-fit models). For low-elevation traits, numDF = 1, denDF = 18. At high-elevation, numDF = 1, denDF = 96. See Fig. 4 for visualization of these results. Variables with n.s. were not included in the best-fit model and therefore do not have a selection gradient to report. * p < 0.10, ** p < 0.05, *** p < 0.01

|  | Low-elevation | | | High-elevation | | |
| --- | --- | --- | --- | --- | --- | --- |
| Trait | z-value | p-value | Selection gradient (β) | z-value | p-value | Selection gradient (β) |
| Field plant height | **3.008** | **0.003** | **+0.638***** | **7.28** | **>0.001** | **+0.288***** |
| Genotypic mean plant height | **2.812** | **0.005** | **+0.359***** | n.s. | n.s. | n.s. |
| Genotypic leaf lobing plasticity | **-2.145** | **0.032** | **-0.263**** | **-1.84** | **0.065** | **-0.075*** |
| Field flowering time | n.s. | n.s. | n.s. | **-5.86** | **>0.001** | **-0.283***** |
| Genotypic leaf area plasticity | n.s. | n.s. | n.s. | **3.16** | **0.002** | **+0.116***** |
| Genotypic mean leaf lobing | n.s. | n.s. | n.s. | **2.84** | **0.004** | **+0.088***** |
